# Characterization of Melanoidins and Color Development in Dulce de Leche, a Confectionary Dairy Product With High Sucrose Content: Evaluation of pH Effect, an Essential Manufacturing Process Parameter

**DOI:** 10.3389/fnut.2021.753476

**Published:** 2021-11-11

**Authors:** Analía Rodríguez, Patricia Lema, María Inés Bessio, Guillermo Moyna, Cristina Olivaro, Fernando Ferreira, Luis Alberto Panizzolo

**Affiliations:** ^1^Departamento de Ciencia y Tecnología de Alimentos, Facultad de Química, Universidad de la República, Montevideo, Uruguay; ^2^Facultad de Ingeniería, Instituto de Ingeniería Química, Universidad de la República, Montevideo, Uruguay; ^3^Laboratorio de Carbohidratos y Glicoconjugados, Departamento de Química Orgánica, Facultad de Química, Universidad de la República, Montevideo, Uruguay; ^4^Laboratorio de Espectroscopía y Fisicoquímica Orgánica, Departamento de Química del Litoral, CENUR Litoral Norte, Universidad de la República, Paysandú, Uruguay; ^5^Espacio de Ciencia y Tecnología Química, CENUR Noreste, Universidad de la República, Tacuarembó, Uruguay

**Keywords:** Maillard Reaction, dairy, dulce de leche, melanoidin, process parameters, pH

## Abstract

The effect on color of the initial pH employed in dulce de leche (DL) production was evaluated through physicochemical and spectroscopical characterization of the melanoidins formed in the process. Melanoidins originated at pH values of 6.5, 7.0, and 7.5, and they were released by the enzymatic hydrolysis of the protein backbone and purified by gel filtration. They showed a significant degree of polydispersity, in general, with molecular weights (MWs) below 1,800 Da. DL produced at a higher pH released melanoidins with higher average MW after the enzymatic hydrolysis. They also presented darker colors (dE^*^ab, C^*^), more closely resembling those typical of the commercial product. Analysis of the fractions isolated by gel filtration using HPLC-DAD and multinuclear NMR showed an heterogeneous and complex composition. Even though structurally related, the 1H NMR spectra of melanoidins showed a higher degree of aromaticity at higher pH values. In conclusion, the pH employed in DL production affects the amount and structure of the colored products originated by MR reactions, and thus the color of the final product.

## Introduction

Dulce de leche (DL) is a brownish, viscous concentrated dairy product with a high sucrose content. Its organoleptic properties, mainly due to the occurrence of the nonenzymatic browning Maillard Reaction (MR), are highly valuated by consumers. DL is an extremely popular confectionary product, particularly in South America. In Uruguay alone the industrial production of DL reaches 7,000 tons per year. Taking also into account informal production, which is estimated to reach comparable levels, leads to a yearly consumption of nearly 4 kg per capita (1). DL is consumed in a variety of ways, including pastry and bom bom filling, toast and cookie spread, and ice cream preparation (2).

The characteristic color of DL is due to the presence of melanoidins, the final products of the MR which can reach concentrations of up to 1.6 ± 0.1 g/100 g (3). It goes from dark brown to light cream-colored, being a commercially relevant characteristic since it frequently determines consumer acceptability (4).

The initial steps in the production of DL include a pH adjustment to neutrality or slightly basic conditions in a range that avoids protein precipitation or the generation of unacceptable flavors. These conditions favor the occurrence of the MR reaction and the generation of products responsible of flavor and color (5–10).

Since many of the reactions involved in the MR have acid–base catalyzed mechanisms, color development is highly dependent on the initial pH, even for a given formula and a set of elaboration conditions. Furthermore, and although the glycation reaction that leads to the Amadori products in the first stage of the MR can occur in acid or basic conditions, the process is favored by basic environments (11).

The pH has a critical role following the formation of the Amadori product. Under acidic conditions, subsequent steps of the MR proceed *via* 1,2-enolization, whereas 2,3-enolization predominates at alkaline pH values (12). Furthermore, sugar fragmentation that occurs at neutral or basic conditions in the MR can lead to melanoidins with different structures compared to those formed at acidic pH values (13).

As the development of the different routes of the MR are highly influenced by the conditions, in particular pH, it is possible that the chemical structure of the resulting melanoidins, and thus the product color, is influenced by the initial pH conditions. In the present work, we studied the effect of the initial pH (pH_i_) on the amount and nature of the melanoidins in DL and the resulting color of the product. This work is based on the methodologies developed from our previous work on melanoidins from DL (14).

The aim of this work was to evaluate the effect of pH on the formation of melanoidins and the development of color in the DL. We tried to answer whether the color differences in DL elaborated at different pH_i_ values are due to the amounts of melanoidins produced and/or variations in their structures.

## Materials and Methods

### Experimental Design

Three production batches of DL were carried out in duplicate at pH_i_s of 6.5, 7.0, and 7.5. The three assayed pH_i_s are within the common pH range used in DL production. Samples were taken at 0, 20, 40, 80, and 150 min after the initiation of the preparation.

The DL preparation was performed according to Rodríguez et al. (14) by concentration of a solution of sucrose (20% w/v) in pasteurized milk with a protein content of 2.9%−3.0% and fat content below 0.1% in a 60-L industrial kettle. The pH_i_ of 6.5 is obtained solely from dissolution of sucrose in milk, and the higher pH_i_ values were adjusted by the addition of sodium bicarbonate. In all cases, pH was monitored with a pH meter equipped with a penetration electrode (Hanna HI8424, HANNA Instruments, RI, USA).

### Color Measurement

Colorimetric measurements of DL were performed in cylindrical white cells (28 mm diameter, 4 mm high). The CIELab color system was used to characterize the samples with the parameters L^*^, a^*^, b^*^, and C^*^. The measurements were performed in a Minolta CM 508d spectrophotometer (Minolta Co. Ltd., Osaka, Japan) with an illuminant D65, a 10° standard observation angle, and specular component excluded. Color differences were measured as ${\text{dE}}_{\text{ab}}^{*}$ = (dL*^2^ + da*^2^ + db*^2^)^1/2^, using milk and water as blanks for DL and melanoidins, respectively.

### Fractionation of DL Into Soluble and Insoluble Water Fractions

**Figure 1:**
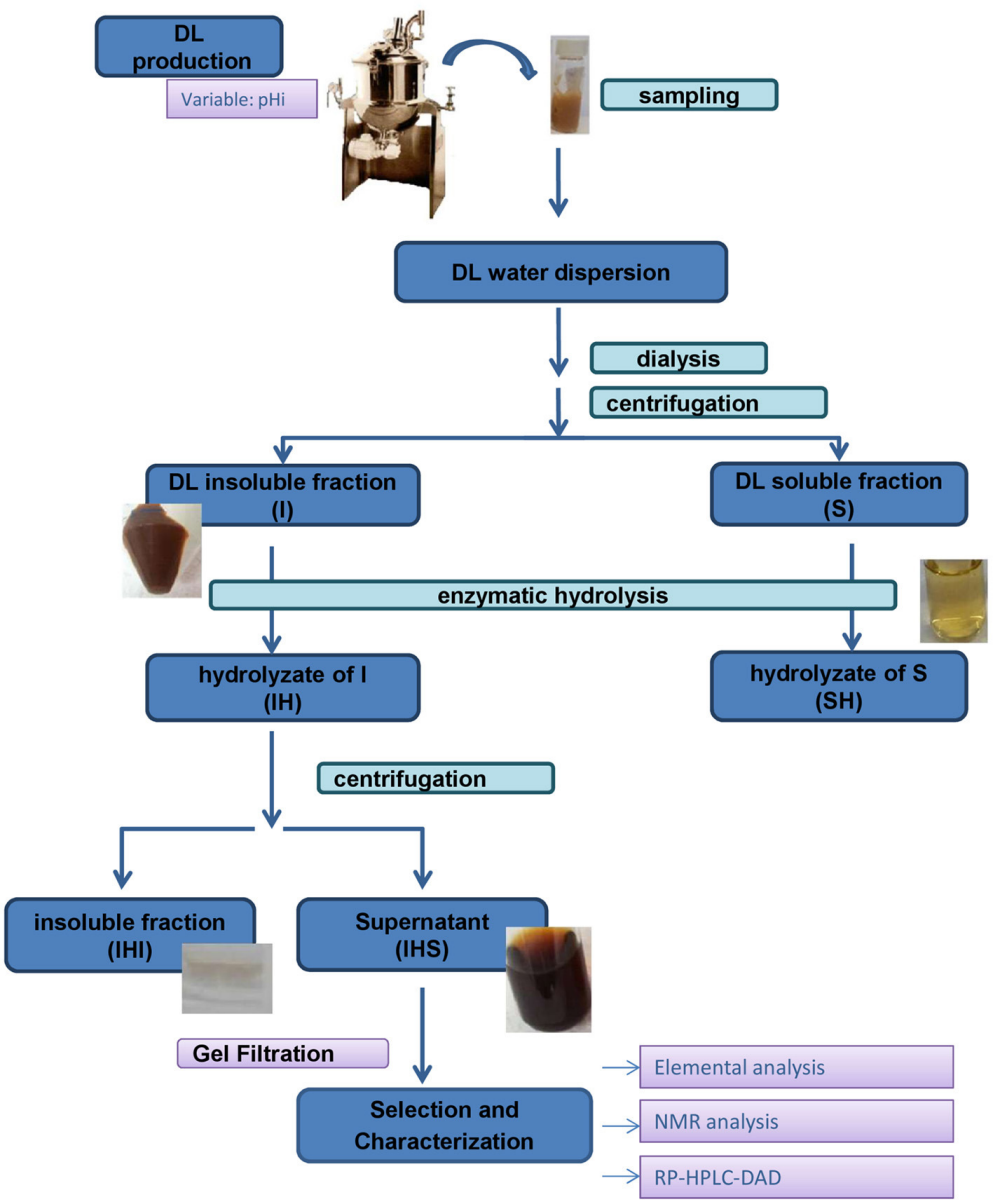
Schematic representation of the isolation and fractionation of DL melanoidins.

The extraction and fractionation of melanoidins were mainly performed as described in Rodríguez et al. (14). Briefly, a water suspension of DL was extensively dialyzed against water to remove sucrose and other low molecular weight (MW) components using a membrane with a cut-off of 6–8 kDa (Spectra/Por, Spectrum Laboratories Inc., Rancho Dominguez, CA, USA). The macromolecular components of DL were then fractionated by centrifugation at 5,500 rpm for 20 min at 25°C, and the supernatant was collected and freeze-dried to give the soluble component (S). The resultant precipitate (macromolecular water insoluble fraction) was washed with water, centrifuged, and freeze-dried to give the insoluble material (I, Figure 1).

The soluble and insoluble percentage, %S and %I, respectively, of the macromolecular fraction of DL was calculated as the percent ratio of the mass of the S and I components relative to their sum. The average value was calculated from the results of three repetitions.

### Enzymatic Hydrolysis of the Soluble and Insoluble DL Macromolecular Fractions

The I and S macromolecular fractions were suspended in 50 mM phosphate buffer at pH 7.0 to a final concentration of 20 mg/mL and treated with 40 UI/mL of Pronase E from *Streptomyces griseus* (4 UI/mg, Sigma-Aldrich, St. Louis, MI, USA) at 37°C for 24 h with magnetic stirring, and then centrifuged at 4,500 rpm for 30 min at 4°C. The supernatants obtained from the treatment of the I and S fractions were separated by centrifugation, freeze-dried, and labeled IHS and SH, respectively (Figure 1) (14).

### Isolation of Melanoidins by Gel Filtration

The melanoidins from the IHS and SH fractions were isolated by gel filtration using a BioGel P2 column (2.6 × 50 cm, BioRad, Hercules, CA, USA). Aqueous acetic acid (0.2% v/v) at a flow rate of 0.6 mL/min was used as the mobile phase, and the eluent was continuously monitored with a Shimadzu RID-6A refractive index (RI) detector. Aliquots of IHS or SH (50 mg in 2 mL of mobile phase) were loaded onto the column, and the fractions were collected every 5 min. The absorbance at 280 and 420 nm of each fraction was recorded on a Pharmacia Ultrospec 1000 spectrophotometer (Pharmacia, Uppsala, Sweden) (14).

The chromatographic separation procedure was repeated three times for each sample to produce enough material for further characterization. Melanoidin fractions corresponding to a nominal MW range from 400 to 1,800 Da were pooled.

### Elemental Analysis

Elemental analyses of the samples (2 mg each) were performed to estimate the nitrogen/carbon ratio (N/C) and sulfur content using a Carlo Erba EA1108 elemental analyzer (Sabadell, Spain).

### NMR Analysis

Selected melanoidin gel filtration fractions were analyzed by NMR. Samples were dissolved in D_2_O containing a trace of sodium 2,2,3,3-*d*_4_-3-(trimethylsilyl)propionate (TSP) used as the internal standard, and transferred to 5-mm tubes (NE-HL5-7, New Era Enterprises, Newark, NJ, USA). Spectra were recorded at 25°C on a Bruker AVANCE III 500 NMR spectrometer (Bruker Corp., Billerica, MA, USA) operating at ^1^H and ^13^C frequencies of 500.13 and 125.76 MHz, respectively, and equipped with a *z*-gradient TXI probe. Water-suppressed ^1^H spectra were recorded using a 30° pulse and presaturation during a 2-s repetition delay, accumulating a total of 256 scans. HSQC spectra were obtained with a gradient-enhanced pulse sequence (15), using 128 scans per slice and a repetition delay of 1 s. ^1^H and ^13^C chemical shifts (δ_H_ and δ_C_, respectively) are reported in ppm.

### HPLC-DAD Analysis of Melanoidins

As described in Rodríguez et al. (14), methanol suspensions of selected melanoidin fractions (6 mg/mL) were stirred in an orbital shaker for 1 h and then centrifuged at 10,000 rpm for 20 min. The supernatant was collected, the solvent was evaporated under a nitrogen stream, and the residue was dissolved in aqueous methanol (5% v/v, 200 μL). These solutions (20 μL) were analyzed by HPLC using a Dionex Ultimate 3000 HPLC (Thermo Scientific, Waltham, MA, USA), equipped with a C18 Zorbax Eclipse column (250 × 4.6 mm, 5 μm particle size) and a DAD detector. A gradient of methanol and water at a flow rate of 1 mL/min was used, going from 5 to 35% methanol in 30 min, and up to 100% methanol at 42 min.

### Determination of Colored Compounds Bound and Not Bound to Protein

Colored compounds that were bound and not bound to protein were determined on the final product (processing time 2.5 h) according to Morales and van Boekel (16) with some modifications. Each sample was analyzed in triplicate.

For determination of colored compounds not bound to protein, DL (2.0 g) was mixed with aqueous TCA (24% w/v, 2.0 mL), homogenized, and centrifuged for 10 min (12,000 g, 25°C). The supernatant was removed and diluted as necessary to read the absorbance at 420 and 550 nm. The blank was a sample of the initial mixture of milk and sucrose, prior to the heat treatment (sample at time 0), and was submitted to the treatment described above.

The browning index was defined as BI = (Abs 420 – Abs 550 nm)/g, after corrections by dilution. The browning index in this case corresponds to colored compounds not bound to proteins (BI_nb_).

Colored compounds bound to protein were released by proteolysis. Briefly, DL (2.0 g) was suspended in 50 mM phosphate buffer (2.0 mL, pH 7.0), homogenized, and treated with a solution of Pronasa E (2 mL of 1 % w/v in 50 mM phosphate buffer, pH 7.0) from *S. griseus* (Sigma Aldrich, ~4 UI/mg). The enzymatic treatment was carried out in capped tubes for 24 h in a water bath (37°C) with agitation (100 rpm). The reaction mixture was then centrifuged at room temperature (12,000 g, 10 min). The supernatant was diluted as necessary to read the absorbances at 420 and 550 nm, and similarly as described above, the browning index corresponding to the total colored compounds was calculated as BI_tot_ = (Abs 420 – Abs 550 nm)/g (bound and no bound to protein).

Colored compounds bound to protein were then calculated as BI_b_ = BI_tot_ – BI_nb_, and the amount of colored compounds bound and not bound to protein were calculated as percentage: (BI_nb_/BI_tot_) × 100 and (BI_b_/BI_tot_) × 100, respectively.

### Statistical Analysis

Analysis of variance (ANOVA) with Tukey tests were carried out to determine significant differences between values (α < 0.05) using Infostat (version 2016, Grupo Infostat, Facultad de Ciencias Agrarias, Universidad Nacional de Cordoba, Argentina). Results were expressed as mean ± SD, and different letters indicate significant differences when α < 0.05.

## Results and Discussion

### Effect of pH_i_ in Color Development and Insoluble Fraction Formation

The effect of pH_i_ on DL color development along the production process showed that higher pH favors an earlier development of darker colors, and thus, probably, the formation of the MR products (Supplementary Figure 1).

The effect of pH on the advancement, and the preferred mechanism of reaction of the MR has been extensively studied (17). Higher pH appears to favor the degradation of sugars, leading to reactive species that speed up the reaction, and the generation of MR products (12). Thus, it cannot be determined *a priori* whether the increase in color is due to a higher concentration of colored compounds or to structural differences of these compounds due to the different mechanisms favored by a specific pH.

**Figure 2:**
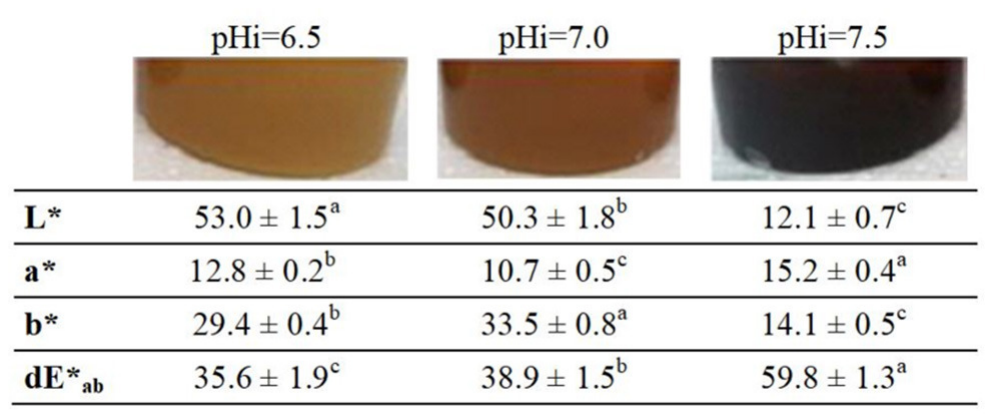
Color parameters of DL elaborated at different pH_i_ values. Results are expressed as mean ± SD of two production batches. Different letters indicate significant differences between groups (α ≤ 0.05).

As observed in Figure 2, the color and color parameters of DL were noticeably dependent on the pH_i_, even for a relatively narrow pH range of 1.0 unit.

The highest pH_i_ produced lower luminosity (L^*^) and a higher color development (dE^*^ab) in the product compared with the initial mix of milk and sucrose.

As we reported previously (14), the semisolid DL dispersed in water can be fractionated in water-soluble (S) and water-insoluble (I) fractions, both colored, but it was observed that the fraction I contributed the most to the color of the product. This was evaluated for DL prepared at three different pH_i_ values, and it was found that the protein-bound colored products were the most abundant based on proteolysis and absorbance measurement (16) (Table 1).

**Table 1:** Protein-bound and free-colored products of the DL prepared at different pH_i_.

**pHi**	**Protein-bound colored products (%)**	**Free colored products (%)**
7.5	98.50 ± 0.03^a^	1.50 ± 0.03^c^
7.0	98.23 ± 0.03^b^	1.77 ± 0.03^b^
6.5	97.63 ± 0.37^c^	2.37 ± 0.37^a^

*Results are expressed as mean ± SD (n = 3). ANOVA analysis was performed by column using Tukey test to state significant differences*.

*Different letters indicate significant differences (p < 0.05) between values in the same column*.

The I fraction yield was evaluated for the different pH_i_ values. As expected, pH_i_ can influence protein interactions and also the elaboration time. In addition since salt removal can affect protein stability and cause aggregation, the I fraction yield was evaluated before and after dialysis.

The pH_i_ did not significantly affect (α ≤ 0.05) the proportion of the I fraction formed during DL elaboration. Indeed, the I fraction before and after dialysis went, approximately, from 10 to 30% when pHi increased (or decreased) from 6.5 to 7.5 (Supplementary Figure 2).

Furthermore, the I fraction before dialysis was almost insignificant when milk and sucrose were initially mixed. This was independent of pH_i_ even at production times of 60 min, despite a brown color that developed for the highest pH_i_. After dialysis, the percentage of I fraction was higher for the initial mixture and the DL, suggesting that salt concentration plays an important role in macromolecule stability (data not shown).

These results show that the formation of the I fraction in DL takes place at an advanced elaboration time, independent of color development and pH_i_. We can assume that the formation of the I fraction is due to the thermal treatment that induces protein associations or the formation of aggregates due to denaturation, disulfide bonds formation, gelling, and even precipitation (18), and where the MR can also contribute through crosslinking (19–21). Lack of a direct correlation between color development and protein insolubilization was reported in the case of ovalbumin (20), but it has not been described before for DL production.

### Color Contribution of the S and I DL Fractions

**Figure 3:**
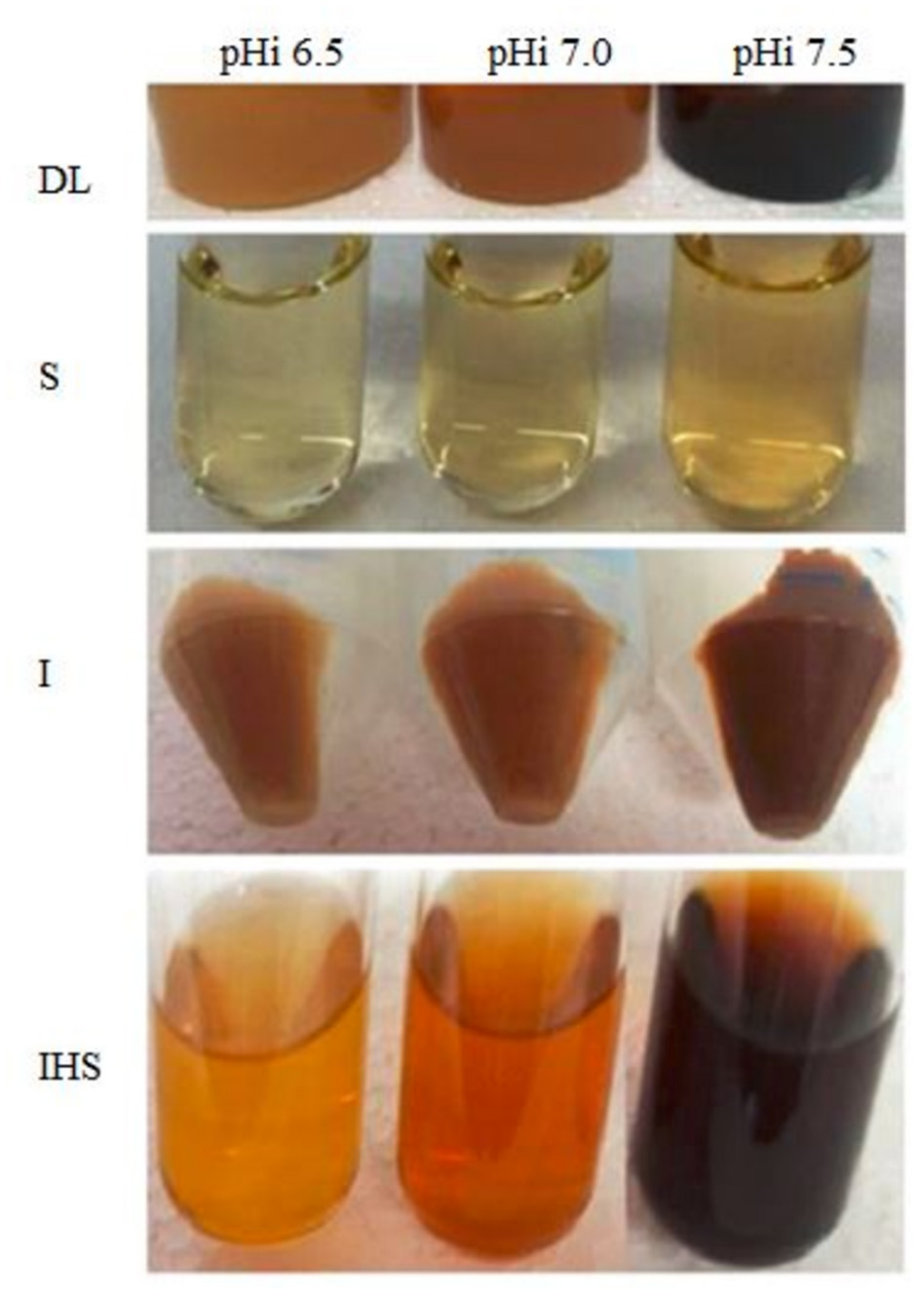
Color of DL prepared at different pH_i_ values and the respective fractions obtained from each product.

The color of the different fractionation components of the DL produced at different pH_i_ values, *vis* the I fraction, the S fraction, and the insoluble fraction after enzymatic hydrolysis of the DL (IHS), are shown in Figure 3. The influence of pH_i_ can be seen in the remarkable differences between the water-soluble colored compounds (S) and the highly colored soluble fraction obtained after hydrolysis (HIS).

It can be observed that the color contribution of the insoluble I fraction (I) is released in a soluble form after hydrolysis (IHS), whereas the remaining insoluble residue remains colorless. This indicates that the main-colored components may be attributed to melanoproteins in I, which released water-soluble melanoidins after enzymatic hydrolysis (IHS fraction).

The color of lyophilized fractions was affected due to the incident light on the powder. To avoid this effect, the color was measured in the corresponding aqueous solutions to obtain the parameters da^*^, db^*^, dC^*^, and dE^*^ab. Higher da^*^, db^*^, dC^*^, and ${\text{dE}}_{\text{ab}}^{*}$ were obtained for SH and IHS. As higher pHi of DL is produced, we can conclude that fraction solutions that contain the soluble melanoidins (SH and HIS) contain the main components responsible for the color differences between the three DL (Supplementary Figure 3).

Regarding the results above, we can conclude that melanoidins could be liberated by enzymatic proteolysis from the melanoproteins that compose the insoluble macromolecular colored components, and this colored solution correctly represents DL color.

### Isolation of Melanoidins in DL by Gel Filtration and MW Profile

**Figure 4:**
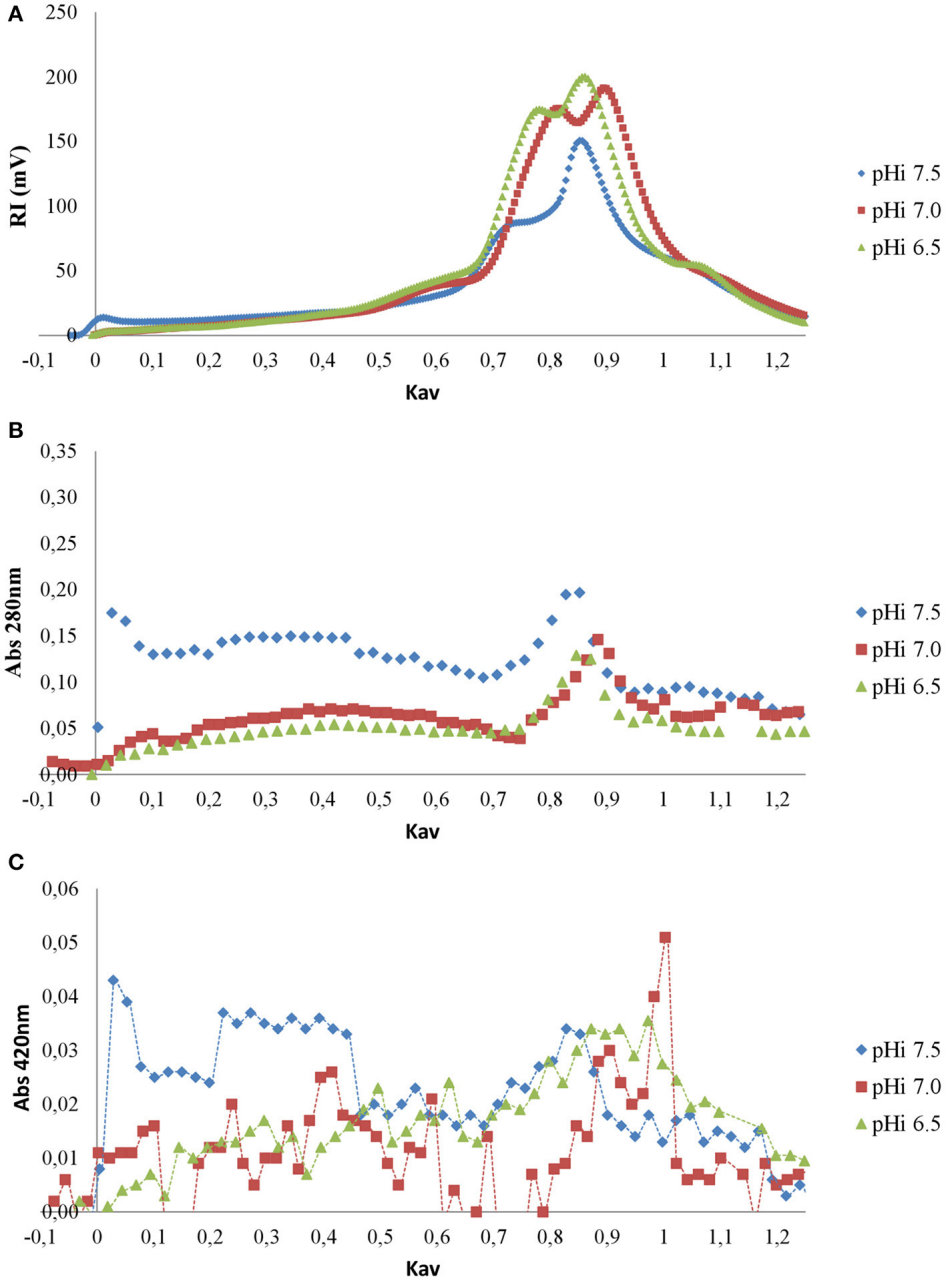
Gel filtration chromatograms of IHS fractions for DL prepared at different pH_i_ values obtained with RI detection **(A)**, absorbance at λ 280 nm **(B)**, and absorbance at λ 420 nm **(C)**.

Soluble melanoidins could be isolated and their MWs could be estimated by gel filtration. The use of an in-line RI detector permits the evaluation of the mass abundance of the different fractions since the detector response is independent of the nature of the solute for sufficiently diluted solutions. Figure 4 shows the chromatograms of the IHS fractions of the DLs prepared at different pH_i_ values.

The chromatogram obtained with RI detection reveals similarities between the IHS from DLs elaborated at pH_i_ values of 6.5 and 7.0 (Figure 4A). On the other hand, the IHS from DL elaborated at a pH_i_ of 7.5 presented a major peak at K_av_ = 0.85–0.90, representing a higher amount of compounds with MW ≥ 500 Da (K_av_ ≤ 0.6), and also compounds eluting with the dead volume (K_av_ = 0) corresponding to a nominal MW ≥ 1,800 Da. These compounds also had higher absorbance at 280 and 420 nm (Figures 4B,C, respectively), and may explain the more intense color of DL prepared at a higher pH_i_.

For further studies and characterization, we selected the melanoidins fractions with K_av_ between 0.17 and 0.65, corresponding to nominal MW from 400 up to 1,800 Da, thus avoiding interference from amino acids or sugars in peaks II and III.

**Figure 5:**
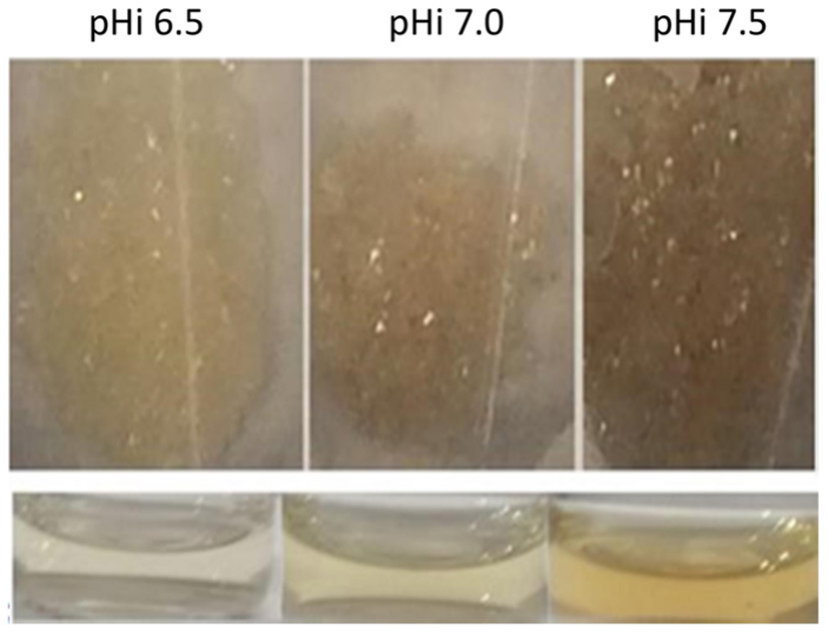
Lyophilized **(top)** and in solution **(bottom)** melanoidins fractions with nominal MW from 400 up to 1,800 Da for the three DL prepared at different pH_i_ values.

The color difference of the melanoidins fractions can be seen in Figure 5. The color parameters of the selected fractions correlate with those of the corresponding IHS and the original DL, and their dependence on pH_i_ (Supplementary Figure 4).

### Elemental Analysis and N/C Ratio of DL Melanoidins

The nitrogen (N) and carbon (C) content of the different DL fractions and melanoidins were determined by elemental analysis, and the N/C ratios were calculated (Table 2).

**Table 2:** Elemental composition of the melanoidin fractions from the three DL prepared at different pH_i_ values^*^.

**pH_**i**_**	**Melanoidin**					**I**	**IHS**
	**% N**	**% C**	**% H**	**% S**	**N/C ratio**	**N/C ratio**	**N/C ratio**
6.5	11	43	8.5	0.66	0.26	0.27	0.29
7.0	11	42	8.3	0.54	0.25	0.28	0.29
7.5	11	42	8.3	0.60	0.26	0.25	0.26

* *The analysis was performed once on pooled material or fractions*.

The N/C ratio obtained for the I fraction and the melanoidins are similar to those found in the studies of the incorporation of sugar into caseins (N/C ratios from 0.23 to 0.25) (22). However, the N/C ratios determined for simple model systems diverge, with N contents of 6% in high MW melanoidins obtained in model systems of glycine with glucose, fructose, or HMF (23), and in glucose–glycine model systems (24). These differences between DL and the model systems are likely due to the different reactivity of macromolecules in the real systems with respect to the models.

The N/C ratio of the insoluble fraction and the melanoidins are very similar, and range from 0.54 to 0.66% (Table 2). Sulfur (S) was detected in the isolated melanoidins from DL produced at different pH_i_ values. S-containing compounds could originate by the MR in the presence of cysteine or cystine, giving rise to thiazolines or thiazolidines among other types of compounds. These are expected to be in higher concentration in melanoidins, explaining the presence of sulfur in these samples (25).

According to Hayashi and Namiki (13), the N/C ratio of melanoidins can increase when the fragmentation route is favored at basic pH. However, the N/C ratio for the fractions was very similar, regardless of the pH_i_ value. This similar N/C ratio can be due to the narrow pH_i_ range tested, close to neutrality. Therefore, the elemental composition would not explain the difference in the color of melanoidins, and so these differences may be better explained by structural differences such as the degree of unsaturation and aromaticity.

### NMR Analysis of the Melanoidin Fractions

**Figure 6:**
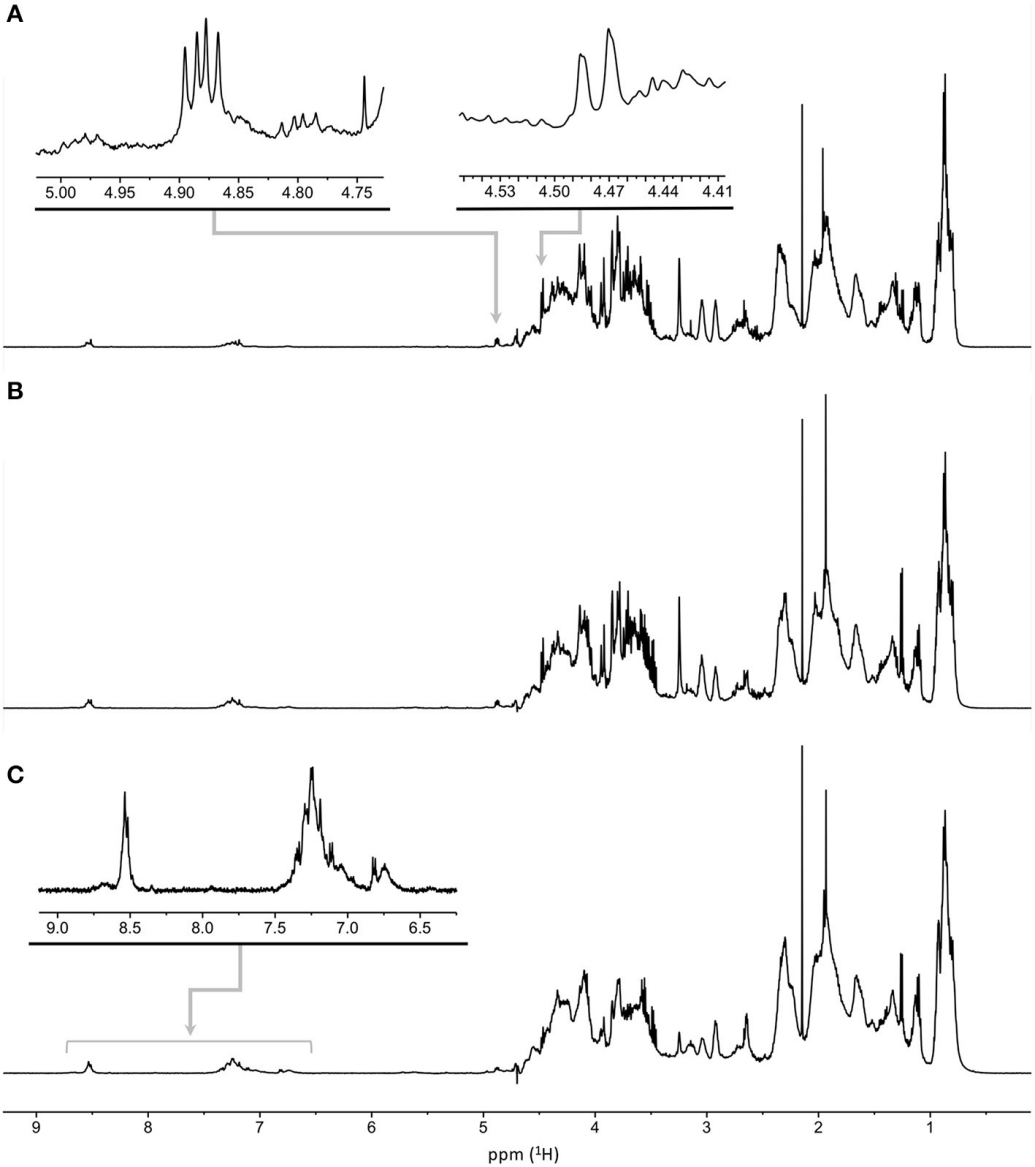
Water-suppressed ^1^H NMR spectra of melanoidins from DL produced at pH_i_ of 6.5 **(A)**, 7.0 **(B)**, and 7.5 **(C)**. The spectral regions employed in the estimation of chemical group ratios are annotated.

**Figure 7:**
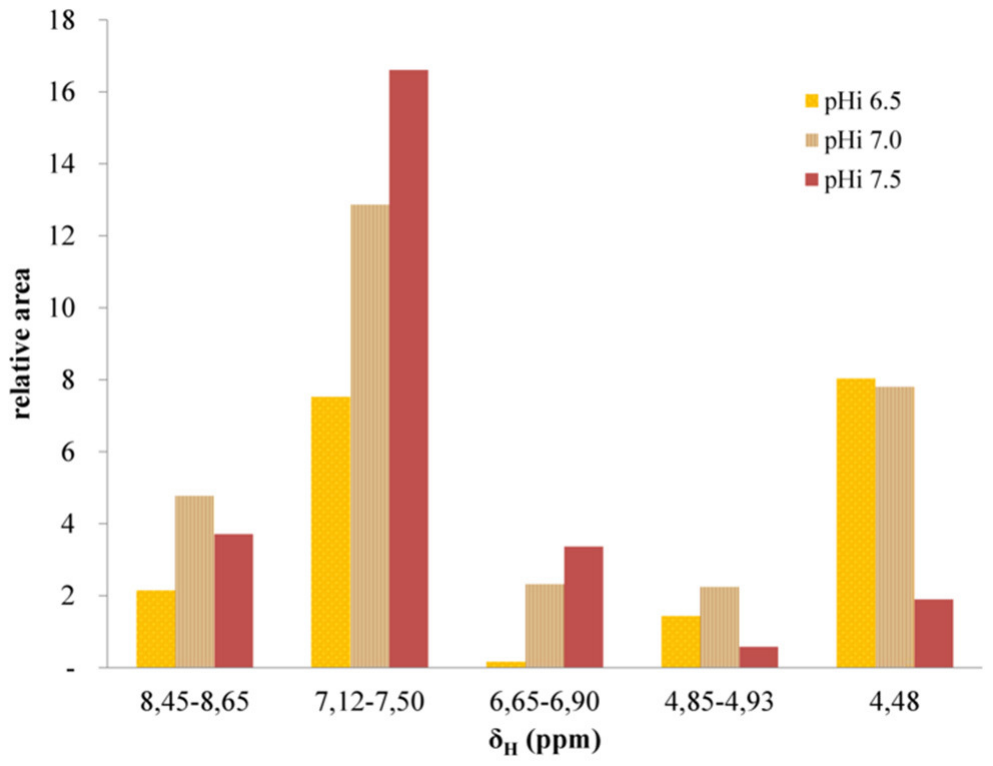
Relative areas of ^1^H NMR signals for the indicated δ_H_ ranges for melanoidins obtained from DL prepared at different pH_i_ values. The area of the aliphatic spectral region (δ_H_ 0.5–2.5) was used as an internal reference.

As recently reported by our group (14), the NMR spectra of melanoidins reveal their structural complexity. Despite isolation and purification, they include a mixture of products comprising initial to end products of the MR, many of which can exist as stereoisomers (26, 27). However, we can partially characterize the chemical groups associated with the potential products of the MR, allowing us to compare melanoidin fractions of DL elaborated at different pH_i_ through a preliminary structural characterization (Figures 6, 7 and Supplementary Figure 5). To do this, we compared the variations observed between the resonances from aromatic protons (δ_H_ 6.65–6.90, 7.12–7.50, and 8.45–8.65), those from protons bound to carbons bearing heteroatoms (δ_H_ 4.93, 4.85, and 4.48), and those from aliphatic protons (δ_H_ 0.5–2.5).

As shown in Figure 6, the ^1^H NMR spectra of the melanoidins fractions from DL elaborated at different pH_i_ values have a similar profile, with only minor differences in the regions of interest. The ratios between the different chemical groups were measured using the spectral regions detailed above, using the area of the aliphatic protons as an arbitrary reference (Figure 7).

The ratio of aromatic to aliphatic protons increases as the pH_i_ in DL elaboration rises. This is consistent with the fact that heteroaromatic, including derivatives of pyrrole, furane, or pyridine, are typical intermediaries, and the final products of the MR and their formation are favored in basic environments (12, 28–30).

**Figure 8:**
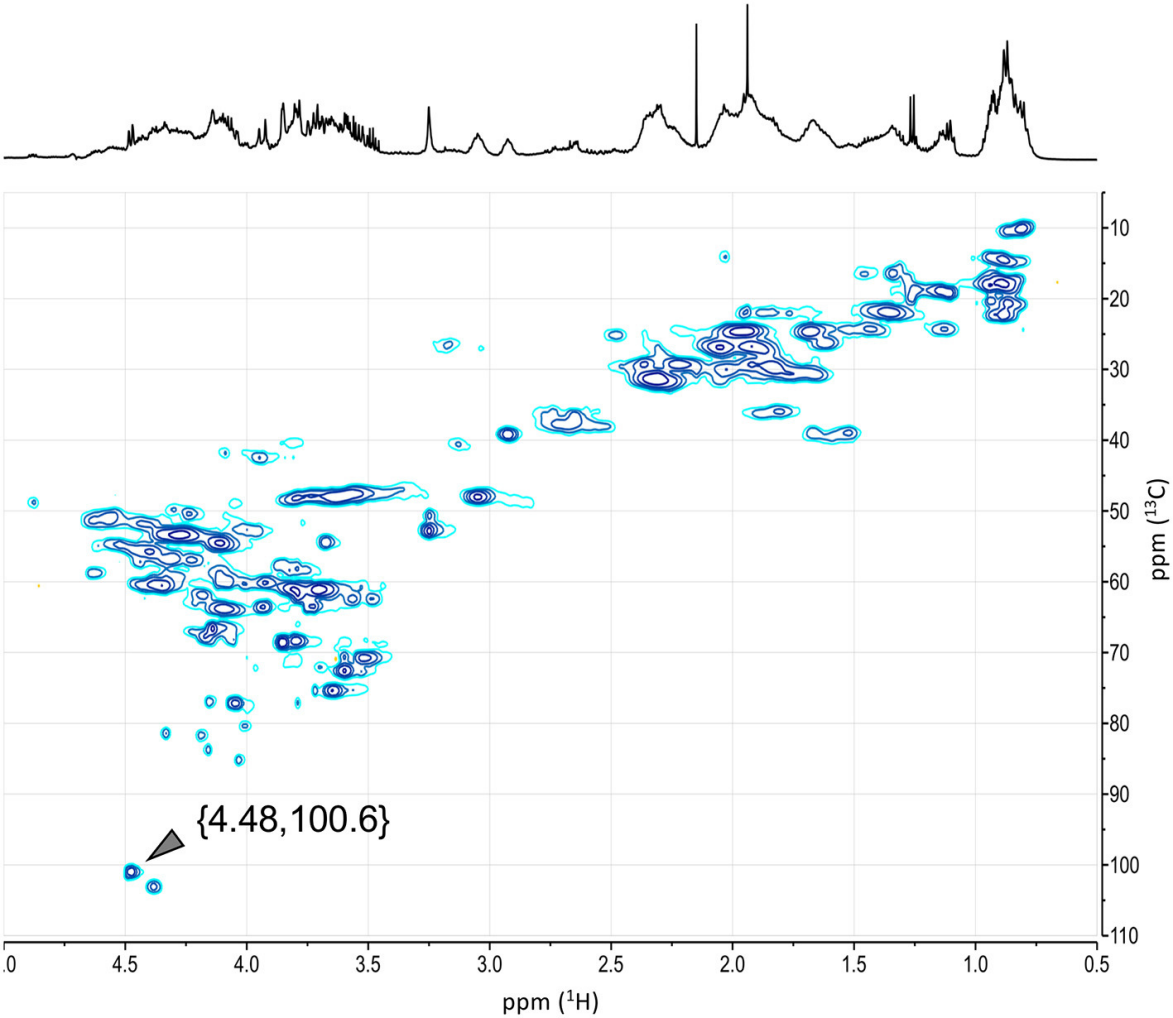
HSQC spectrum of melanoidins from DL prepared at pH_i_ 7.0. The ^1^H-^13^C correlation corresponding to imines from Amadori condensation products is annotated. Similar results were observed at higher and lower pH_i_ values (see Supplementary Figure 5).

As we have recently reported (14), the formation of Amadori products from the reaction between milk proteins and sugars can be evidenced in the HSQC spectra of the melanoidin fractions. Indeed, the one-bond correlation between the proton resonance at δ_H_ 4.48 and the carbon signal at δ_C_ 100.6 corresponds to the anomeric center of the reducing end of β-d-glucopyranosyl residue of lactose after condensation with the ε-amine group of a lysine residue (Figure 8). Imine condensation is followed by the formation of the Amadori products and subsequent reactions where the galactose residue is preserved in the 1-deoxyosone and 3-deoxyosone routes, which affect only the glucose residue at the reducing end of lactose. On the other hand, the transformation of the Amadori product *via* the formation of a 4-deoxyosone leads to the loss of a galactose residue. As shown in Figures 7, 8, the signals of the anomeric proton are larger at lower pH_i_ and almost undetectable at higher pH_i_, indicating that different routes could predominate at different DL production pH_i_ values. The hypothesis that the galactose residue in disaccharides can be lost or transformed is further supported by the proposals of Pischetsrieder et al. (21, 31), which suggest that at neutral to basic pH the Amadori product degradation is *via* the 4-deoxyosone route with galactose residue loss.

### Analysis of Melanoidins by HPLC-DAD

**Figure 9:**
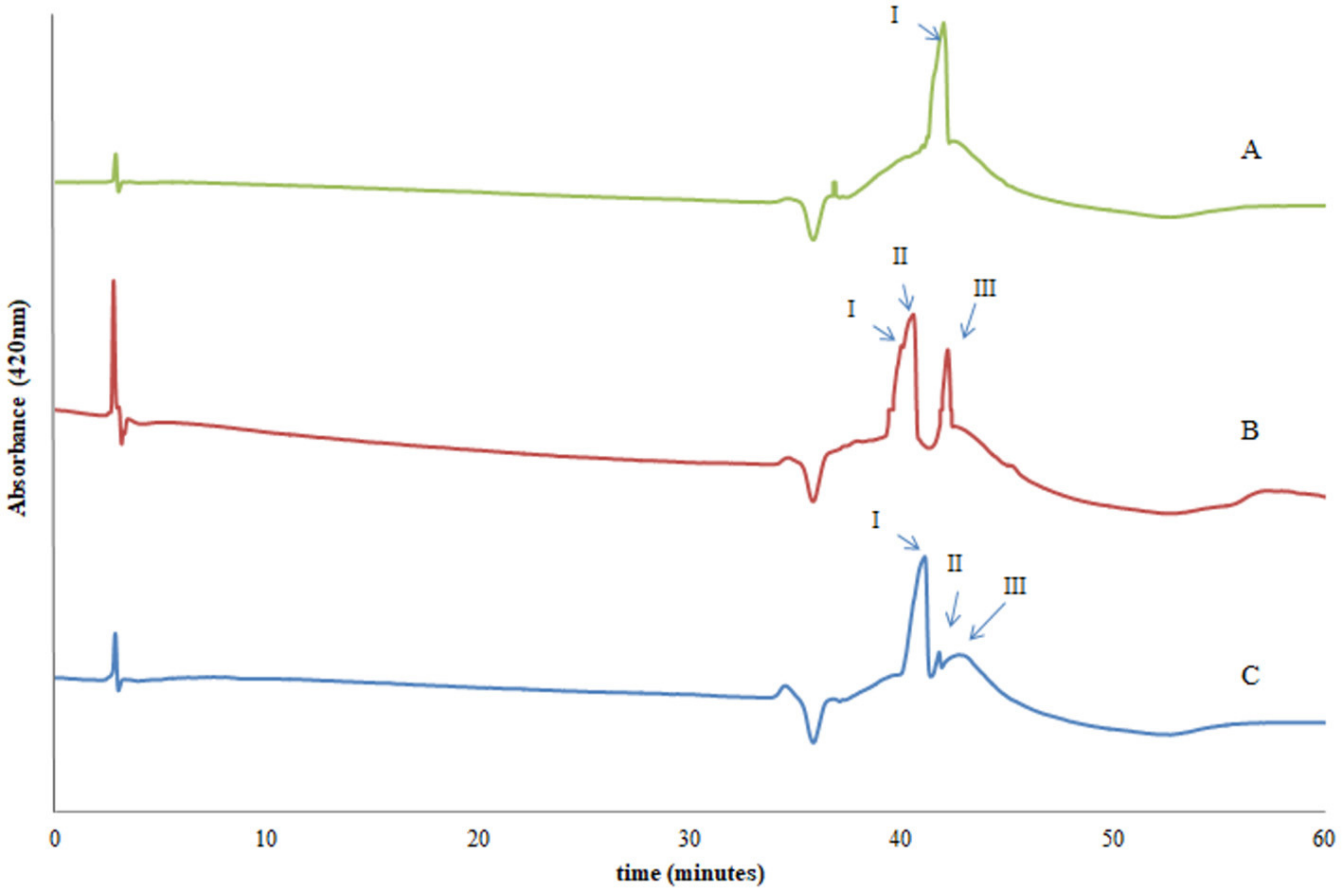
HPLC-DAD chromatograms at λ 420 nm of melanoidins (nominal MW from 400 to 1,800 Da) from DL prepared at pH_i_ of 6.5 **(A)**, 7.0 **(B)**, and 7.5 **(C)**.

The HPLC chromatograms of the melanoidins fractions with a MW from 400 to 1,800 Da obtained from DLs elaborated at different pH_i_ with detection at 420 nm are shown in Figure 9.

While the chromatograms are relatively simple, some peaks are not completely resolved due to broadening. It has been proposed that this is due to the presence of polymeric compounds which lowers the resolution when this chromatographic system is employed (MeOH/H_2_O gradient) (32).

The hydrophobic character of the melanoidins results in higher retention times relative to the noncolored aromatic compounds present in the mixture, indicating their higher hydrophobicity (14). These results were observed regardless of the pH_i_ used in DL preparation (Supplementary Figure 6). This behavior is compatible with the formation of structures with a higher degree of condensation and aromaticity as advanced MR products are formed. This observation is also consistent with the analysis of the ^1^H NMR data of melanoidins.

The chromatographic profiles of melanoidins are simpler for products obtained from DL produced at the highest and lowest pH_i_ assayed, showing the presence of one or two main compounds. On the other hand, the melanoidins from DL produced at an intermediate pH_i_ are more complex. As previously discussed, this may reflect that at more acidic or basic pH_i_ values some reaction pathways are favored, whereas at pH_i_ 7.0 several pathways leading to melanoidin formation may occur with a similar probability.

The UV–Vis spectra of the compounds eluting in the major peaks annotated in Figure 9 for the melanoidins obtained from DL elaborated at pH_i_ 7.0 present absorbance bands in the UV and, in particular, the visible regions. Peak I presented two maxima in the visible region at λ 537 and 594 nm, peak II presents maxima at λ 562 and 592 nm, and peak III presents three maxima at λ 553, 592, and 599 nm (Supplementary Figure 7). Similar spectra were obtained for the corresponding peaks in the chromatograms of the melanoidins obtained from DL prepared at pH_i_ 6.5 and 7.5 (data not shown).

From the evaluation of the UV–Vis spectra it can be inferred that although the compounds may be structurally similar, they likely correspond to different degrees of advancement of the MR, and thus confer different color to the sample.

## Conclusions

Melanoidins in DL are found forming high MW melanoproteins, mainly in the water-insoluble component that is formed at advanced stages of processing in the analyzed pH_i_ range. The main colored compounds released by enzymatic hydrolysis had low MW ≤ 1,800 Da and were more abundant in the melanoproteins from DL prepared at higher pH_i_. The melanoidins isolated from DL with nominal MW from 400 to 1,800 Da presented higher color parameters (${\text{dE}}_{\text{ab}}^{*}$, C^*^), as did the corresponding DL. The elemental composition was very similar and not conclusive, but through the combined use of the NMR and HPLC-DAD analysis, it was found that the colored compounds that developed, even though structurally similar, present a higher degree of aromaticity in DL prepared at higher pH_i_. Therefore, it can be concluded that the greater or lesser color of the DLs obtained at different pH_i_ is not exclusively due to a greater or lesser concentration of the melanoidins, but also to structural differences according to the production processing parameters of DL. To gain a detailed insight on the structure of these compounds, it will be necessary to isolate the different melanoidins components and perform an exhaustive structural characterization by spectroscopic methods like MS and NMR.

## Data Availability Statement

The original contributions presented in the study are included in the article/Supplementary Material, further inquiries can be directed to the corresponding author/s.

## Author Contributions

AR: conceptualization, methodology, investigation, writing original draft and review & editing, and visualization. PL: conceptualization and supervision. MB: methodology, investigation, and validation. GM: investigation and writing review and editing. CO: investigation and visualization. FF: conceptualization, methodology, validation, investigation, resources, writing original draft and review & editing, supervision, and project administration. LP: conceptualization, methodology, resources, writing review and editing, supervision, project administration, and funding acquisition. All authors contributed to the article and approved the submitted version.

## Funding

This work was supported by a PhD grant for AR from the Agencia Nacional de Investigación e Innovación (ANII, award POS_NAC_2012_1_8813). Support from the Programa de Desarrollo de las Ciencias Básicas (PEDECIBA) is acknowledged as well.

## Conflict of Interest

The authors declare that the research was conducted in the absence of any commercial or financial relationships that could be construed as a potential conflict of interest.

## Publisher's Note

All claims expressed in this article are solely those of the authors and do not necessarily represent those of their affiliated organizations, or those of the publisher, the editors and the reviewers. Any product that may be evaluated in this article, or claim that may be made by its manufacturer, is not guaranteed or endorsed by the publisher.
