# Supplementary material for: Characterization of Melanoidins and Color Development in Dulce de Leche, a Confectionary Dairy Product With High Sucrose Content: Evaluation of pH Effect, an Essential Manufacturing Process Parameter
Source: Front Nutr. 2021 Nov 11;8:753476. doi: 10.3389/fnut.2021.753476 (PMC8632489; doi:10.3389/fnut.2021.753476)
Supplement: Supplementary file 1 [file Data_Sheet_1.pdf]

Characterization of melanoidins and color development on a confectionary dairy product with high sucrose content. Evaluation of pH effect, an essential manufacturing process parameter

Analía Rodríguez, Patricia Lema, María Inés Bessio, Guillermo Moyna, Cristina Olivaro, Fernando Ferreira, and Luis Alberto Panizzolo

Supplementary Material

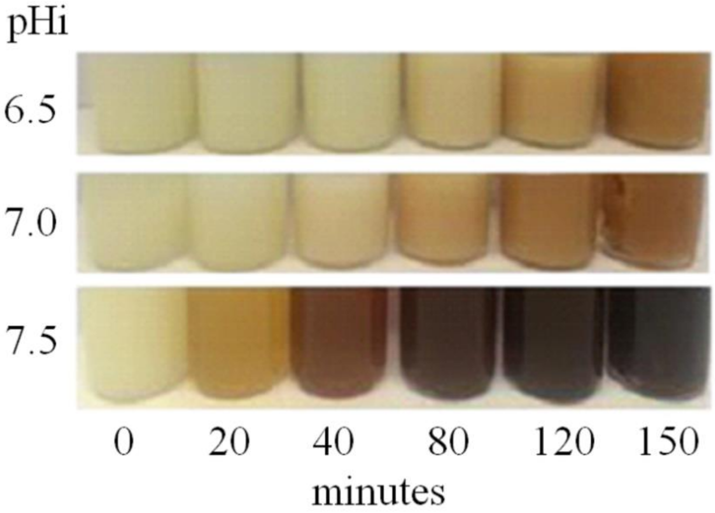

Supplementary Figure 1. Effect of pH_i on color development during DL production.

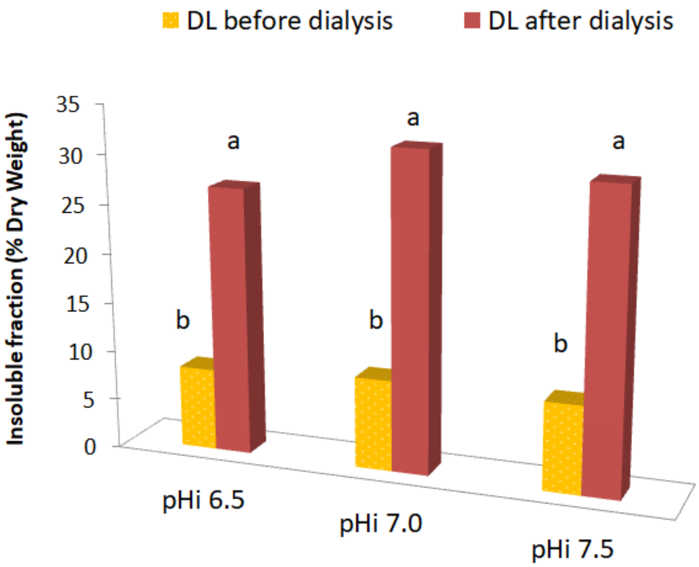

Supplementary Figure 2. Insoluble fraction percentage of DL prepared at three different pH_i values. Different letters indicate significant differences between groups ($\alpha \leq 0,05$)

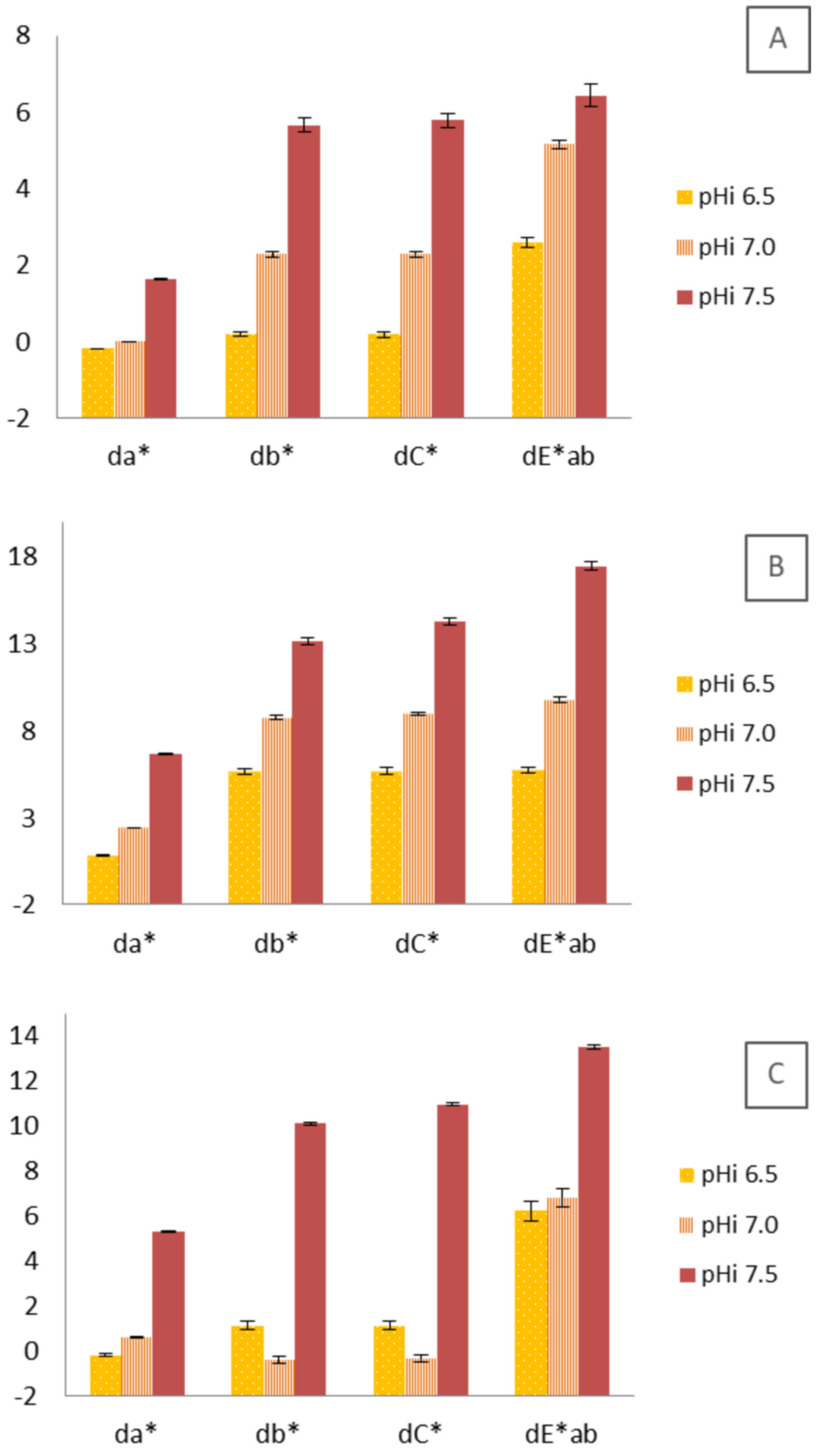

Supplementary Figure 3. Color parameter of DL prepared at different pH_i values and its main colored fractions (SH and IHS). SH (A), IHS (B) and DL (C).

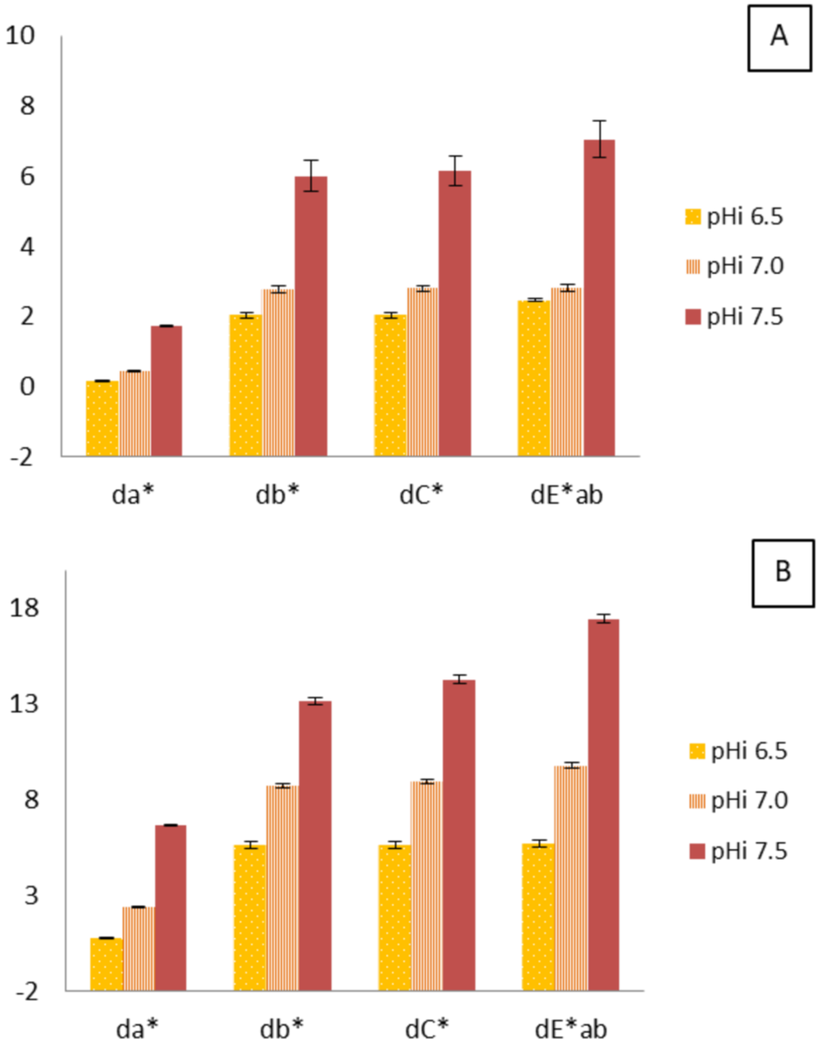

Supplementary Figure 4. Colour parameters of melanoidins (A) and IHS fractions (B) for DL prepared at different pH_i values.

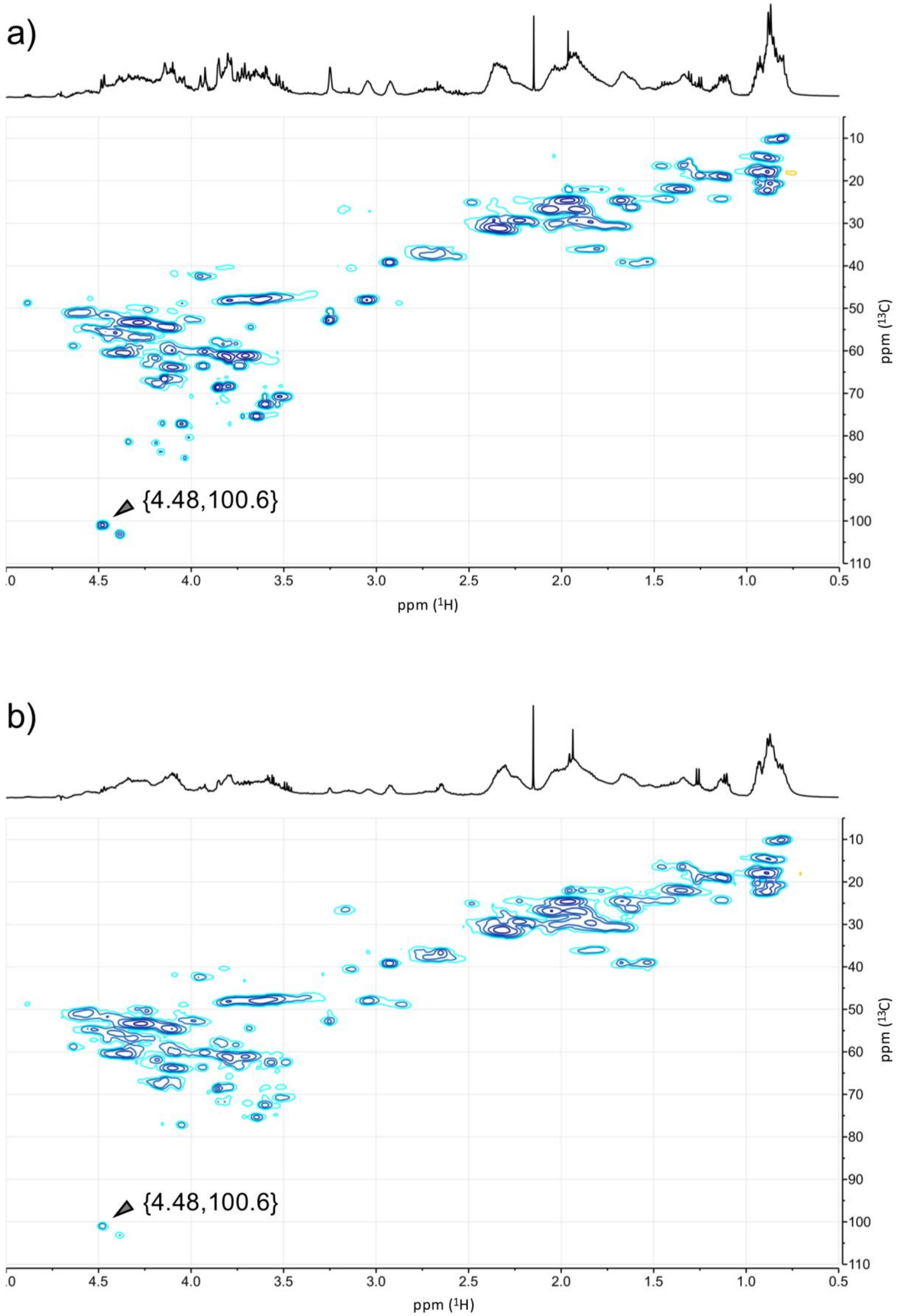

Supplementary Figure 5. HSQC spectra of melanoidins from DL prepared at pH_i 6.5 (a) and 7.5 (b). The ^1H-^{13}C correlation corresponding to imines from Amadori condensation products is annotated.

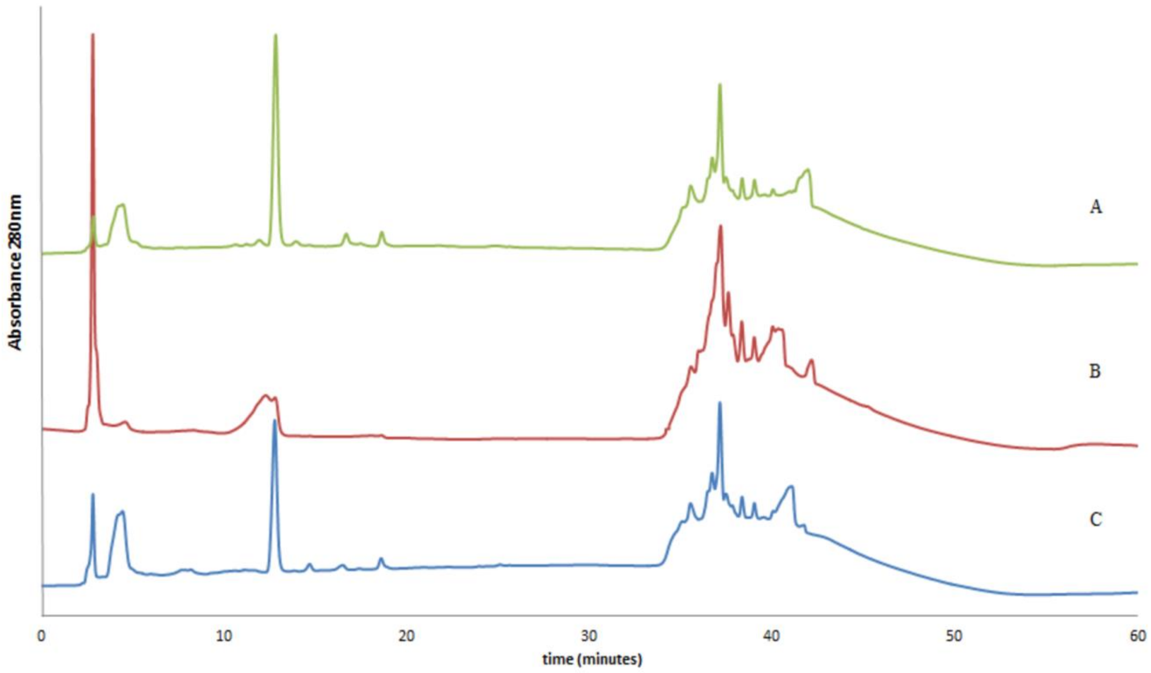

Supplementary Figure 6. RP-HPLC chromatograms at λ 280 nm of melanoidins with nominal MW from 400 to 1,800 Da from DL prepared at pHi 6.5 (A), 7.0 (B) and 7.5 (C).

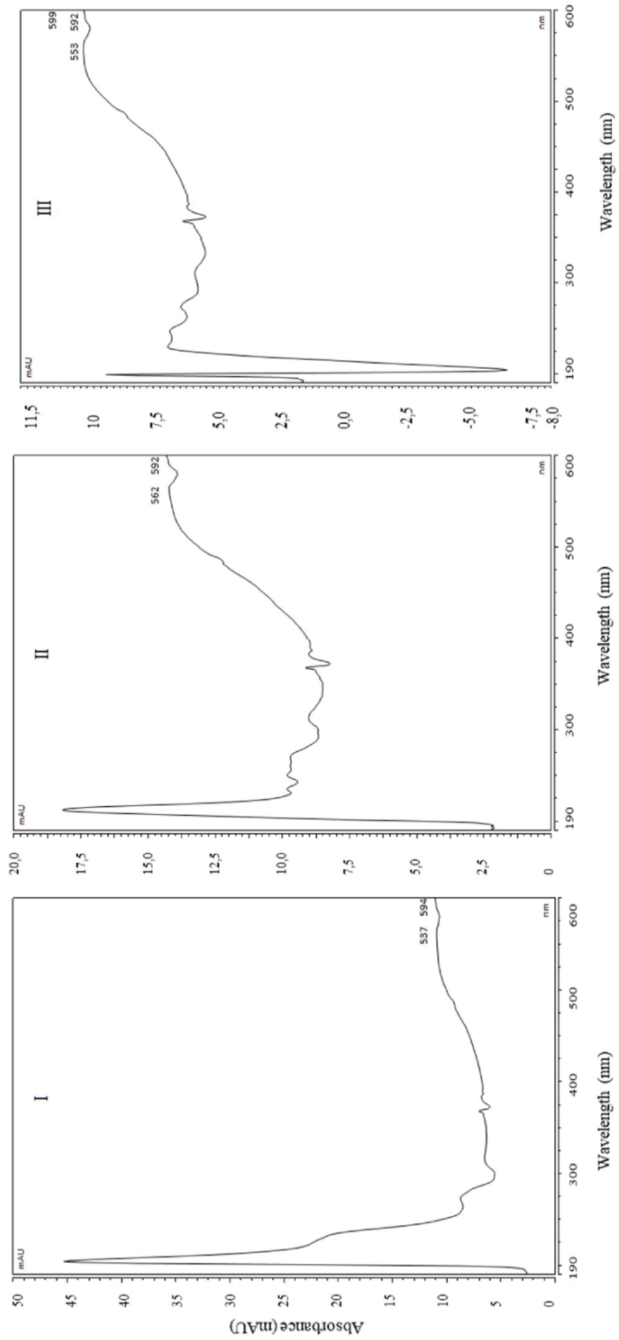

Supplementary Figure 7. UV-Vis spectra of peaks I, II and III of the HPLC-DAD chromatograms described in **Figure 9.**
